# Effect of wet ageing with different marination methods on quality characteristics of Korean native black goat meat

**DOI:** 10.5713/ab.260362

**Published:** 2026-09-01

**Authors:** Shine Htet Aung, Rupasinghe Ramesh Nimantha, Phyo Htet Htet Kyaw, Oni Faith Oluwaseyi, Madiththe Gedara Asela Sandaruwan Abeyrathna, Young-Sun Choi, Ki Chang Nam

**Affiliations:** 1 Department of Animal Science and Technology, Sunchon National University, Suncheon, Korea; 2 Department of Animal Science, University of Veterinary Science, Nay Pyi Taw, Myanmar; 3 Jeollanam-do Agricultural Research and Extension Services, Gangjin, Korea

**Keywords:** Goat Meat, Injection, Meat Quality, Pineapple Solution, Soaking, Wet Ageing

## Abstract

**Objective:**

This study evaluated the effects of wet ageing combined with soaking or injection marination using pineapple powder solution on the physicochemical properties of Korean native black goat meat.

**Methods:**

The samples were marinated with pineapple solution using soaking or injection methods, vacuum-packed, and wet-aged at 4°C for 21 days, with analyses performed at 7-day intervals. Physicochemical characteristics, lipid oxidation, texture profile, protein degradation, and fatty acid composition were analyzed.

**Results:**

Wet ageing combined with pineapple powder solution marination reduced pH and improved tenderness, as indicated by increased myofibrillar fragmentation index and reduced hardness (p<0.05). However, marination also reduced water-holding capacity and increased cooking loss, with the injection treatment showing greater tenderization than soaking. Microbial counts remained within acceptable ranges throughout storage and were lower in the marinated treatments than in the control during later ageing stages. Additionally, soaking enhanced polyunsaturated fatty acid content and improved the nutritional quality indices by reducing the calculated atherogenicity and thrombogenicity indices.

**Conclusion:**

Injection marination with pineapple powder solution into wet-aged goat meat may improve tenderness, while soaking provided a more favorable fatty acid profile during wet ageing.

## INTRODUCTION

The World Health Organization has reported that the global population aged over 65 years is expected to increase substantially by 2050. Ageing is often associated with an increased risk of noncommunicable diseases and nutritional challenges, highlighting the importance of adequate intake of high-quality protein sources. Goat meat is considered a lean meat option with high protein content and has potential as a value-added meat product [1]. However, goat meat consumption may be limited by quality-related characteristics, particularly its relatively tough texture. Therefore, improving tenderness and physicochemical properties through appropriate processing strategies may contribute to the development of more acceptable goat meat products.

Post-mortem ageing is a typical post-harvest process that improves meat quality traits such as tenderness, juiciness, and flavor through the muscle structure breakdown of endogenous enzymes [2]. The ageing process can be carried out by either dry-ageing (aerobic conditions for placing unpackaged under controlled chilled temperature, relative humidity, and airflow) or wet-ageing (anaerobic conditions for placing the vacuum packaged) [3]. There are certain negatives to dry-ageing, including the fact that the process takes more time and space, and results in more weight loss from water loss in the cuts, more trim loss, and more shrinkage [4]. In contrast, wet ageing is more frequently used in the meat industries since it costs less time, investment, and effort and the resultant produces less loss trimming and has a higher water retention rate [5].

Wet ageing is a process that involves storing vacuum-sealed meat at a set temperature under anaerobic conditions. During wet ageing, the meat becomes more tender as a result of the myofibrillar protein being degraded by endogenous enzymes. The flavor of meat can also be improved after a period of ageing due to the release of flavor-associated substances. Endogenous enzymes have an initial impact on the biochemical and physical alterations in meat that occur during the ageing process, followed by bacterial enzyme activity [6]. In addition, the quality attributes of ageing meat are also improved by modifications such as marination.

The marination process involves soaking or injecting the meat with a flavorful liquid including ingredients such as wine, herbs, soy sauce, brine, spices, and tenderizers. Soaking is the process of submerging the meat for a set amount of time in the marinade to allow the flavors to permeate the meat [7]. Injecting is a marination method where marinade is injected directly into the meat using a syringe or meat injector without the time losses involved in immersion [8]. The texture, flavor, and overall quality of meat are significantly influenced by marinades with tenderizing effects, such as enzyme activity and acidity that stimulate proteolysis. Pineapple powder contains proteolytic enzymes together with organic acids and other constituents that may influence meat quality [9], which may modify muscle structure properties.

However, there is little research on the physicochemical quality changes of wet-aged goat meat with different marination methods. Therefore, this research aimed to modify the physicochemical quality of meat by wet ageing and to evaluate the effects of different marination methods on the quality characteristics of Korean native black goat meat (*Capra hircus coreanae*) during wet ageing.

## MATERIALS AND METHODS

### Raw materials and the wet ageing process

A total of 36 Korean native black goats (*Capra hircus coreanae*) were used in this study. The goats were raised under uniform feeding and husbandry conditions at a commercial goat farm in Gangjin-gun, Jeollanam-do, Korea. The goats were 16-month-old castrated males with an average live body weight of 26.56±2.50 kg. Longissimus dorsi (*M. longissimus dorsi*) muscles were collected at 24 h postmortem and transported to the laboratory in oxygen-permeable packaging under refrigerated conditions before being stored at 4°C.

All visible connective tissue and external fat were removed before marination. The loin muscles were cut into approximately 200 g portions. For the soaking treatment, 300 mL of pineapple powder solution (1:7, w/v) per kilogram of meat was added to plastic containers (10 cm depth×40 cm width), and the meat samples were soaked for 3 h, with the samples turned after 1.5 h to ensure uniform marination. For the injection treatment, 300 mL of pineapple powder solution (1:7, w/v) per kilogram of meat was injected using a multi-needle injector. A pineapple powder solution was prepared by dissolving 100% pure commercial pineapple powder (Saemaeul) in distilled water at a ratio of 1:7 (w/v). The solution was homogenized until completely dissolved and used immediately for marination.

After marination, the samples were vacuum-packaged and wet-aged at 4°C for 21 days. Twelve goats were assigned to each treatment (control, soaking, and injection). From each treatment, meat samples from three different animals were allocated to each ageing period (1, 7, 14, and 21 days), resulting in three independent biological replicates for each treatment× ageing combination (n = 3). Each meat sample was analyzed only once at its assigned ageing period.

### Proximate composition

Moisture content was determined by measuring weight loss after oven drying at 104°C for 24 h. For pH measurement, 2 g of sample was homogenized with 18 mL distilled water, filtered, and the filtrate was measured using a calibrated pH meter. The method of Uttaro et al. [10] was used for water-holding capacity (WHC) measurement with a few modifications. Briefly, a centrifuge (Combi 514-R; Hanil) was used to centrifuge a 5 g sample of ground meat for 10 min at 168×g. The sample’s weight was then determined. The WHC was calculated according to the following equation:


(1) $$WHC\;(\%)=\frac{\left(W_{s}-W_{f}\right)}{W_{s}}\times 100$$

where *W_s_* represents the initial sample weight and *W_f_* represents the final weight of the sample after centrifugation and removal of released water. After centrifugation, the exudate was carefully removed using filter paper before weighing the remaining meat sample.

The sample’s cooking loss (CL) was calculated as a percentage weight loss after 90 sec of cooking on a double-sided electric grill pan until the meat sample’s internal temperature reached 72°C using standardised sample cuts (30×50×10 mm). The color values (CIE L*, a*, b*) were measured on the meat surface using a calibrated colorimeter (CR-410; Minolta) after blooming at room temperature for 30 min.

### Lipid oxidation

The lipid oxidation of wet-aged meat was evaluated by measuring the malondialdehyde (MDA) concentration with the 2-thiobarbituric acid reactive substance (TBARS) method [11]. The ground meat sample (5 g), distilled water (15 mL), and butylated hydroxytoluene (7.2% in ethanol, w/v) solution (50 μL) were mixed in a 50 mL centrifuge tube and homogenized. The mixture (2 mL) and 4 mL of acid/trichloroacetic acid solution (20 mM TBA/15% TCA, w/v) was mixed in a 15 mL conical tube and heated in a hot-water bath at 90°C for 15 min. It was then cooled for 15 min with cool water before centrifuging 3 mL of the supernatant at 1,500×g for 15 min. The absorbance value was measured at 531 nm with a spectrophotometer. The blank sample was prepared with distilled water instead of the sample and the measurement of TBARS was expressed in milligrammes of MDA per kilogramme of the sample.

### Microbial analysis

For microbial analysis, 10 g of sample was homogenized with 90 mL of sterile saline (0.85% NaCl) in a blender (LED Embossing Stomacher; Bnf Kore) for 2 min and serially diluted. Appropriate dilutions were plated on the surface of dry film culture media (3M Petrifilm Aerobic Count Plates for total aerobic bacteria enumeration and 3M Petrifilm Coliform Count Plates for coliform enumeration), incubated at 37°C for 24 h, and results were expressed as log CFU/g.

### Texture profile analysis

Marinated meat samples (100±5 g) were cooked in a 95°C water bath until the internal temperature reached 80±2°C, cooled to 30°C before texture analysis. After cooling, the cooked meat was cut in a manner of 15 mm length and 10 mm width in a ridge following the distance of 20 mm from the probe. Texture profile analysis was performed using a TA-XT2 analyzer with a cylindrical probe (diameter: 50 mm). The pre-test, test, and post-test speeds were 5.0, 1.0, and 5.0 mm/s, respectively. The trigger force was set at 5.0 g, and the interval between the two compression cycles was 2.0 s. Hardness was expressed as kilogram-force (kgf), while cohesiveness, springiness, chewiness, and gumminess were calculated from the force–time curves. Springiness values were expressed as ratios rather than percentages.

### Myofibrillar fragmentation index

The myofibrillar fragmentation index (MFI) value of the meat sample was estimated by using the method of Olson et al. [12], with an MFI buffer (0.1M KCl, 0.02M KH_2_PO_4_, 0.001M EDTA, 0.001M MgCl_2_, and 0.001M NaN_3_). 2 g of sample was mixed with 20 mL of MFI buffer (ice cool) and homogenized at 1,258×g for 30 s. Afterwards, the mixture was centrifuged at 1,500×g for 15 min and removed the supernatant. Once more, the procedure was carried out while the residue was suspended in 20 mL of MFI buffer. The remaining sediment was re-mixed in 10 mL MFI buffer and vortexed, and then the homogenate was filtered through an 18-mesh filter (1.0 mm Test Sieve; ChungGye Sieve). The protein concentration of the myofibrillar suspension was adjusted to 0.5 mg/mL, and the absorbance was measured at 540 nm using a spectrophotometer. The MFI value was calculated by multiplying the absorbance at 540 nm by 200.

### Fatty acids composition

The fatty acid composition of the sample was evaluated with the method of O’Fallon et al. [13]. To separate the fatty acid methyl esters, 0.7 mL of 10 N KOH and 6.3 mL of methanol were added to each 1 g sample. After that, this mixture was placed in a water bath that was kept at a 55°C water bath. The samples were forcefully shaken every 30 min while they were heating. The product was cooled in ice-cold water for two minutes before being treated with 0.58 mL of (24 N) H_2_SO_4_. The combination was heated again under similar conditions, maintaining the same constant temperature. Three millilitres of hexane were added, and the mixture was centrifuged (HANL Combi-514R) for five minutes at 1,500×g before being pipetted into a vial using a Pasteur pipette. Fatty acid methyl esters (FAMEs) were analyzed using a gas chromatography-flame ionization detector (GC-FID, Agilent 7890 series; Agilent Technologies) equipped with an HP-88 capillary column (60 m×0.25 mm i.d.×0.20 μm film thickness; Agilent Technologies). The injector was operated in split mode with a split ratio of 25:1 at 250°C. Helium was used as the carrier gas at a flow rate of 25 mL/min. Hydrogen and air were supplied as detector gases at flow rates of 40 and 400 mL/min, respectively. The FID temperature was maintained at 260°C. The oven temperature was maintained at 220°C during analysis. Fatty acids were identified by comparing retention times with a commercial FAME standard mixture (CRM47885 or SMB00937; Sigma-Aldrich). Peak integration was performed using the Agilent ChemStation software. Fatty acid composition was expressed as the percentage of total identified fatty acids. The atherogenicity index (AI), thrombogenicity index (TI), and hypocholesterolemic/hypercholesterolemic (HH) ratio were calculated according to the following equations:


(2) $$\text{AI}=\frac{\left[\text{C}12:0+4\times (\text{C}14:0)+\text{C}16:0\right]}{\left[\sum \text{MUFA}+\sum \text{PUFA}\right]}$$

(3) $$TI=\frac{\left[C14:0+C16:0+C18:0\right]}{\left[0.5\times \left(\sum MUFA+\sum n6\right)+3\times \sum n3+\frac{\sum n3}{\sum n6}\right]}$$

(4) $$HH=\frac{\left[C18:1cis9+C18:2n6+C20:4n6+C18:3n3+C20:5n3+C22:6n3\right]}{\left[C14:0+C16:0\right]}$$

### Statistical analysis

The experimental unit was the individual goat, and three independent biological replicates were analyzed for each treatment×ageing combination. Data were analyzed using the GLM procedure of SAS software (ver. 9.4; SAS Institute). A two-way analysis of variance (ANOVA) was performed to evaluate the effects of marination treatment (control, soaking, and injection), ageing period (1, 7, 14, and 21 days), and their interaction (treatment×ageing). When significant differences were detected (p<0.05), mean comparisons were performed using Tukey’s multiple comparison test. Statistical significance was declared at p<0.05. In addition, multivariate analysis was performed using MetaboAnalyst 6.0 (https://www.metaboanalyst.ca/). Fatty acid data were normalized by sum normalization and Pareto scaling before partial least squares discriminant analysis (PLS-DA). PLS-DA was used as an exploratory approach to visualize differences among treatment groups.

## RESULTS AND DISCUSSION

### Proximate composition

Table 1 shows the impact of wet ageing with different marinating methods on goat meat quality characteristics during 21 days. The moisture content of the control group was significantly increased during ageing (p<0.05). However, the injection group decreased and the soaking group increased (p> 0.05). The injection and soaking groups were significantly higher than the control group on the initial day, 7 days, and 14 days (p<0.05). After 21 ageing days, the control group observed the highest moisture content followed by soaking and injection (p<0.05). The reduction in moisture content observed during ageing may be associated with moisture migration, drip loss, and structural changes of muscle proteins during storage [14].

The pH of ageing meat changes as part of the natural biochemical processes during ageing. The pH of all the treatments decreased during ageing. Li et al. [15] found that the growth of lactic acid bacteria was the cause of the decreasing pH in vacuum-sealed wet-aged meat. However, the control group increased again after 21 ageing days, but it was lower than the value of the initial day. This could be due to the production of biogenic amine and ammonia by the action of enzymes. The control and injection groups were significantly higher than the soaking group on the initial day (p<0.05). However, the injection groups observed the significantly lowest pH value as the ageing day increased, followed by the soaking and control groups (p<0.05). Holman et al. [16] reported that the ultimate pH continued to increase in the wet-aged grass-fed beef during ageing. Other researchers also mentioned that beef pH increased with wet ageing. According to them, the pH of the wet-aged meat will continue to increase due to the protein breakdown. In the present investigation, the pH of goat meat marinated with pineapple solution decreased even after 21 days of wet ageing. It is possible due to the acidic nature of the pineapple solution, which could impact the pH of wet-aged goat meat rather than the action of proteolytic enzymes.

WHC and CL are important factors that can affect the product’s yield and quality. The control and soaking groups continued to increase the WHC after 21 ageing days (p<0.05) while the injection group remained statistically unchanged (p>0.05). During the ageing, the highest WHC of all treatments was observed on 7 and 14 of the ageing days. The highest WHC was shown in the control group, followed by the soaking and injection groups during wet ageing (p<0.05). The current result is consistent with Kadıoğlu et al. [17], which found that lowering the pH of meat samples can have a considerable impact on the meat’s water-holding ability. Opposite trends in WHC and CL in our current investigations (Table 1). The control group decreased the CL during ageing (p< 0.05), but the soaking and injection groups significantly increased it (p<0.05). The highest CL was observed in the soaking group on the initial day and 7 days of ageing, followed by the injection and control groups (p<0.05). After 14 and 21 days of ageing, significantly higher values were observed in the soaking and injection groups compared with the control (p<0.05). The quantity of water lost from meat while cooking is known as “cooking loss,” and it is mostly caused by denaturing proteins. As proteins break down, there is a loss of structural integrity within the muscle tissue that can result in the disruption of the linkage between myofibrils (contractile units within muscle cells) and the cell membrane [18]. The loss of linkage and structural changes during ageing can promote the migration of water from the muscle cell structure. The WHC and CL are related to the water migration within muscle tissue. In addition, the moisture condition can affect the increase and decrease of CL [19]. In the present study, both soaking and injection marination with pineapple powder solution decreased pH and WHC while increasing CL in wet-aged Korean native black goat meat. Although marination with pineapple solution improved tenderness, it also resulted in increased CL and reduced WHC, moisture content, and pH, indicating a trade-off between tenderness and other quality attributes. Furthermore, injection marination produced greater tenderization than soaking, suggesting more effective distribution of the pineapple powder solution within the muscle. These findings indicate that although pineapple powder solution marination, particularly by injection, enhanced tenderness through increased protein degradation, it also reduced WHC and increased CL. Therefore, tenderness improvement should be considered alongside these changes in water-retention properties.

### Microbial analysis

The various ageing procedures, such as marination, ageing time, and methodologies, might result in environments that are more or less conducive to microbial growth. Some microbial activity may enhance the flavor of meat but excessive activity can lead to deterioration. According to EU rule No. 2073/2005, meat consumers may consume up to 5 log (CFU/g), however, more than 7 log (CFU/g) is not considered safe food [20]. In the current study, total aerobic bacterial counts and coliform counts remained relatively low during the 21-day ageing period; however, these parameters alone cannot fully determine microbiological safety. The TPC and coliform count of all the treatments continued to increase until 14 days of age and decreased again on day 21. The reduction in microbial counts observed at the end of storage may have resulted from multiple factors, including the anaerobic environment created by vacuum packaging, changes in pH, nutrient availability, accumulation of inhibitory metabolites, microbial interactions, and experimental variability. Although reduced oxygen availability in vacuum packaging can influence the growth of aerobic microorganisms [21], oxygen limitation alone cannot fully explain the observed microbial changes because all treatments were vacuum-packaged throughout the storage period. On the initial day, TPC peaked in the control group, while coliform counts were highest in the control and injection groups (p<0.05). On the 7 days of ageing, the highest TPC and coliform count were observed in the control group (p<0.05) but there is no significant difference between soaking and injection (p>0.05). After 14 and 21 days of ageing, the injection group showed the lowest TPC and coliform count followed by soaking and injection groups (p<0.05). These findings indicate that marination with pineapple powder solution reduced total aerobic bacterial and coliform counts compared with the control during refrigerated storage under the conditions evaluated [22]. Furthermore, injection marination showed a greater reduction in microbial counts than soaking, possibly because direct delivery of the pineapple powder solution into the muscle promoted more uniform distribution of pineapple-derived compounds within the meat matrix.

### Color attributes

The lightness (CIE L*) value of all treatments continued to increase during 21 days of ageing (p<0.05) (Figure 1A). On the initial day, the highest CIE L* value was observed in the soaking group followed by the injection and control (p<0.05). In the later ageing process, the injection group showed the highest CIE L* value followed by the soaking and control (p<0.05). Nadzirah et al. [23] also observed that meat with a pineapple juice or bromelain powder treat displayed a higher CIE L*. Kadıoğlu et al. [17] claimed that the meat that has the higher lightness is caused by enlargement of the muscle proteins, variations in light reflection caused by ionic strength, and a decrease in pH.

The wet ageing significantly affected the redness (CIE a*) of the goat meat (p<0.05) (Figure 1B). The decrease in color stability in aged meat is proposed to result from several causes, such as significant mitochondrial damage, metabolite depletion linked to myoglobin redox stability, and increased oxidative stress [24]. However, marination with pineapple solution positively influenced CIE a* value than the control until 14 days of ageing. This could be due to its ability to stabilize myoglobin redox and decrease oxidative stress. A significant difference was detected on day 1, with the control and injection groups exceeding the soaking group (p<0.05). Following 7 days of ageing, the control treatment declined significantly (p<0.05), but the soaking treatment did not differ significantly (p>0.05). However, the CIE a* value of the injection group went up unknownly. On 14 ageing days, the injection observed the highest CIE a* value followed by soaking and control (p<0.05). At the final ageing stage (21 days), the soaking group showed the highest CIE a* value but there was no significant difference with the control group (p>0.05). In contrast, a significant reduction was observed in the injection group (p<0.05). The reduction in redness during ageing may be associated with changes in myoglobin chemistry, oxidation processes, or structural modifications of muscle tissue [25].

During the ageing process, fluctuating yellowness (CIE b*) values were observed in all treatments (Figure 1C). The control group did not significantly change until 14 days of ageing but increased on 21 ageing days (p<0.05). The soaking group didn’t significantly change during ageing. Although the injection group didn’t significantly change during 14 days of ageing, the CIE b* value significantly dropped after 21 days of ageing (p<0.05). Although ageing enhances meat quality, discoloration from metmyoglobin and darkening from melanodin might result in economic losses. The current studies showed that color stability could be maintained for 21 days when wet ageing goat meat using the soaking marinated method with pineapple juice.

### Myofibrillar fragmentation index

The fragmentation of myofibrillar proteins and structure improves meat tenderness throughout ageing, as evidenced by increased MFI. The wet ageing process had a strong effect on the MFI of all treatments in the current research (p<0.05). This could be the result of age-related proteolysis, which is largely brought on by the calpain system and includes things like actin filament and Z-disk weakening, costamere and desmin degradation, and tropomyosin breakdown [26]. A significant difference was maintained during ageing, with the soaking and injection groups exceeding the control (p<0.05). The incorporation of pineapple powder or pineapple-derived products in meat processing has been reported to enhance protein degradation due to their proteolytic activity [27]. The increased MFI observed in the present study may be attributed to the combined effects of endogenous proteolysis during wet ageing and exogenous protease activity from the pineapple powder solution. Because exogenous protease can contribute to myofibrillar protein degradation, pineapple powder solution marination may accelerate structural breakdown and improve tenderness characteristics. In the present study, the injection treatment showed a greater increase in MFI than the soaking treatment, suggesting more effective modification of muscle structure.

### Texture profile analysis

The texture profile study of wet-aged goat meat, including its hardness, springiness, gumminess, chewiness, and cohesiveness, is displayed in Table 2. Based on the present findings, the hardness rapidly and significantly dropped from day 1 to day 7 of ageing, and then it slowed down insignificantly until day 21 of ageing. This decreasing trend coincided with an increase in MFI, much like the hardness trend. Ali et al. [28] reported that the hardness of aged meat decreased as the wet-ageing days increased. The mechanical strength of intramuscular connective tissue decreases with age, mostly as a result of proteolysis brought on by endogenous enzymes, which reduces hardness [29]. During the ageing period, the injection group observed the lowest hardness value followed by the soaking and control groups (p<0.05). Modifying the condition of processing meat, such as through the use of exogenous enzymes, can result in a notable enhancement of its tenderness [30]. The chewiness of meat refers to its textural characteristics related to the resistance and effort required when chewing, often influenced by factors such as muscle fiber composition and connective tissue content [31]. The current observations revealed a substantial link between chewiness and hardness.

In the context of meat, cohesiveness is a textural quality that characterizes how effectively the flesh pieces adhere to one another when chewed. In the current result, the cohesiveness of all treatments increased as the ageing days increased, but the values of the soaking and injection groups were statistically similar (p>0.05). The control group increased significantly after 7 days of ageing (p<0.05). The increased cohesiveness observed after pineapple powder solution treatment may be related to structural modifications of myofibrillar proteins during proteolysis. According to Livesey et al. [32], polypeptides, which include peptides and proteins, can have significantly different stickiness. Springiness varied during the study; however, no significant treatment effect was found (p>0.05). In addition, there was an insignificant difference between the ageing days in the control and injection groups (p>0.05). The springiness of the soaking group was significantly higher on day 7 of ageing (p<0.05) but no significant differences were found across the remaining ageing days. In the context of mechanical texture, springiness refers to the rate and degree to which a material regains its previous shape following deformation by an outside force [33]. The gumminess of all treatments continued to decrease during the ageing period. The lowest gumminess was observed in the soaking group on the initial day but in the soaking and injection groups after 7 days of ageing (p<0.05). In late ageing days, there were statistically similar between all groups, but the soaking and injection had lower gumminess (p>0.05). The addition of a marinating pineapple solution to the wet-ageing process of goat meat had a good effect on reducing hardness as well as chewiness and gumminess. Therefore, elderly people can achieve the desirable level of meat tenderness by using pineapple to regulate wet-aged goat meat.

### Lipid oxidation

The MDA concentration in wet-aged meat was measured as an indication of lipid oxidation with the TBARS method (Figure 2). Lipid oxidation is critical for the quality of ageing meat because it produces unpleasant flavors and harmful substances as a result of excessive oxidation. As the ageing time increased, the TBARS value increased in lamb and goat ageing meat [34]. In the current result, the TBARS values of all treatments decreased on day 7 and continued to increase until 21 days of ageing. This could be due to the highest activation of antioxidant compounds due to endogenous and exogenous enzymes on day 7 of ageing. In addition, the reason for re-increasing the TBARS value after 7 days of ageing could be due to the decreasing activity of antioxidant enzymes (superoxide dismutase, glutathione peroxidase, and catalase) [35]. On days 1, 7, and 14, the injection group had the lowest TBARS value, followed by the soaking and control groups (p<0.05). After 21 days of ageing, the soaking and injection groups did not differ significantly (p>0.05), yet both were significantly lower than the control group (p<0.05). This is possible due to the various antioxidants in pineapple solution, including vitamin C, flavonoids, and phenolic compounds [36]. Additionally, the injection treatment had a greater effect on oxidative stability than the soaking treatment, possibly because direct injection promoted a more uniform distribution of the pineapple powder solution within the muscle.

### Fatty acids composition

Figure 3 shows the multivariate analysis of the effect of wet ageing on the fatty acid profile during the ageing period without considering treatments. The PLS-DA score plot showed distinct clustering patterns among the control, soaking, and injection groups, suggesting differences in fatty acid profiles among treatments (Figure 3A). The variable important projection (VIP) score demonstrated the five fatty acid compositions (n-6/n-3, monounsaturated fatty acids [MUFAs], Oleic acid, polyunsaturated fatty acids [PUFAs], and n-6) that are major differences for discrimination of PLS-DA (Figure 3B). On day 21 of ageing, the n-6/n-3 was at its maximum; the MUFA and oleic acid were at their highest on day 7 of ageing; and the n-6 on day 14 of ageing. In addition, PUFA was the highest on day 1 of ageing. The lowering of PUFAs during ageing may lead to the development of volatile compounds [37]. Utama claimed that the reduction in MUFAs and n-3 PUFAs brought on by an extended ageing period may cause the ratio of n-6 to n-3 to increase [38].

The variations in fatty acid composition during wet ageing can result in distinct flavor and aroma compounds when cooked. The current results showed that the marinating method significantly affected the fatty acid profile of wet-aged goat meat (Table 3). According to the VIP score, saturated fatty acid (SFA) was the highest on day 1 and the lowest on day 7 of ageing. Specifically, the soaking group exhibited the lowest levels of SFA such as myristic acid (14:0) and palmitic acid (16:0) during the 14-day ageing process (p<0.05). However, values were comparable across all treatments after 21 days (p>0.05). The consumption of SFAs is associated with a higher intake of energy but it has been linked to several diseases and cancers these days, most notably coronary heart disease [39]. Especially, myristic acid and palmitic acids may contribute to the development or progression of atherosclerosis and cardiovascular diseases [40]. The highest unsaturated fatty acid (UFA) was observed in the soaking group during 14 days of ageing (p<0.05), all treatments were statistically similar at day 21 (p>0.05). As the unsaturation increases in the fatty acid composition, it can reduce the firmness of the fat tissue due to its low melting point. According to the Wood et al. [39], UFA is more prone to rancidity, which is the cause of several off-flavors and aromas in meat that are referred to as “rancid” and “fishy”.

Day 1 of ageing had the highest amounts of MUFAs in the injection group; days 7 and 14 observed the highest levels in the control and soaking groups. Nevertheless, there is no appreciable change between the groups after 21 days of ageing. Oleic acid (18:1) is the main MUFA that separates the groups from one another. Oleic acid, which lowers blood pressure and modifies insulin resistance among other favorable health consequences in humans, is thought to be the main flavor difference [41]. In addition, Hwang and Joo [42] stated that MUFA and oleic acid have a good correlation with the meat flavor and savory taste.

In addition, the highest PUFA was in the soaking groups on days 1 and 7 of ageing and in the injection group on day 14 (p<0.05), but the values were statistically similar after day 21 (p>0.05). This could be due to the difference in lipolysis activity between marinating methods. Specifically, the high n-3 PUFAs such as linolenic acid (18:3), eicosatrienoic acid (20:3), and eicosapentaenoic acid (20:5) were found in the soaking group, while eicosatrienoic acid and eicosapentaenoic acid were highest in the injection group. Wood et al. [39] reported that the high n-3 PUFA levels led to greater levels of lipid breakdown products, including saturated and unsaturated aldehydes, alcohols, and ketones. In addition, the consumption of eicosapentaenoic acid (20:5) and docosahexaenoic acid (22:6) has favorable health benefits. Meanwhile, linolenic acid (18:3) was the highest in the soaking group on days 7 and 14, but in the control group after 21 days of ageing. Linolenic acid produces more intense odors than oleic and linoleic acids [43]. And then, the researcher claimed that the higher concentrations of linolenic acid, eicosapentaenoic acid, and docosahexaenoic acid in lamb create a higher flavor preference and overall liking for taste panellists. It should be noted that the fatty acid composition was expressed as a relative percentage of total fatty acids rather than as absolute concentrations. Therefore, the observed differences among treatments may have been partially influenced by changes in moisture content following pineapple powder solution treatment.

The nutritional quality indices (PUFA/SFA, n-6, n-3, n-6/n-3, AI, TI, and the proportion of HH of wet-aged goat meat with different marinating methods are shown in Table 4. The PUFA/SFA ratio was influenced by both marination treatment and ageing period (p<0.05). The soaking treatment showed the highest ratio on day 1, whereas the injection treatment reached its highest value on day 14. A ratio below 4 for n-6/n-3 PUFA is recommended, as higher values may increase the likelihood of heart attack and thrombosis [44]. However, the current study was higher than the recommended level. This is because there is not enough n-3 PUFA concentration to balance out high n-6 PUFA, which might result in unfavorable ratios (n-6/n-3). A lower AI is linked to hindering the development of atherosclerosis, while a lower TI is associated with the prevention of thrombosis [45]. A higher ratio of HH suggests more beneficial fats, potentially lowering cholesterol levels [46]. The soaking group had not only the lowest AI and TI but also the highest HH during days 1, 7, and 14 of wet ageing, but there was statistically similar on day 21. The inclusion of lower SFA and higher UFA concentrations in the sample is responsible for those indices. Factors such as temperature, marination duration, mechanical alterations, and ingredient interaction may influence the solubility and mobility of individual fatty acids, thereby causing compositional differences between the soaking and injection groups. According to current studies, consumers, including older people, can improve their PUFA consumption and lower their risk of atherosclerosis, thrombosis, and cholesterol by choosing wet-aged goat meat that has been soaked in pineapple solution. A limitation of this study is the relatively small sample size. Therefore, further studies with larger sample sizes are needed to validate the present findings. In addition, water-injected and pH-matched control treatments were not included in the present study. Therefore, the individual effects of pineapple constituents, acidity, and the physical marination procedures could not be distinguished. Consequently, the observed responses should be interpreted as the combined effects of marination with pineapple powder solution under the conditions of this study.

## CONCLUSION

Wet ageing improved protein degradation, antioxidant activity, and texture characteristics in Korean native black goat meat. Marination with pineapple powder solution enhanced tenderness but also increased CL and reduced WHC, moisture content, and pH, highlighting a trade-off between tenderness improvement and the preservation of other quality characteristics. The soaking treatment showed better color stability during ageing, whereas injection produced greater tenderization. Multivariate analysis indicated changes in fatty acid profiles during ageing, and pineapple powder solution marination influenced the relative fatty acid composition, with soaking generally exhibiting a more favorable PUFA profile. Overall, these findings demonstrate that wet ageing combined with pineapple powder solution marination can modify the physicochemical characteristics of goat meat. However, further studies are needed to optimize processing conditions and validate the underlying mechanisms and potential functional or health-related benefits.

## Figures and Tables

**Figure 1:**
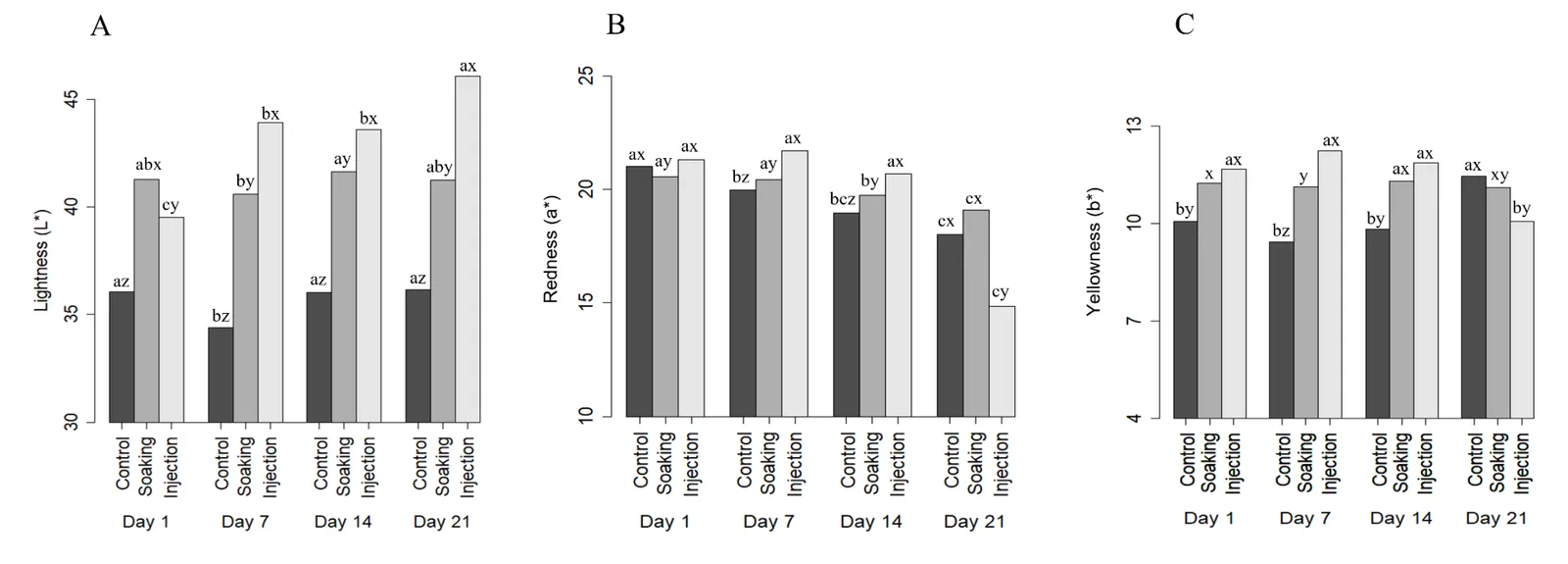
Effect of wet ageing with different marinating methods on color attributes (A) lightness, (B) redness, and (C) yellowness of goat meat during 21 d. Changes in color attributes (lightness [L*], yellowness [b*], redness [a*]) across ageing periods (Days 1, 7, 14, and 21) under Control, Soaking, and Injection treatments. Lightness and yellowness generally increased with ageing time, with Injection showing higher values at later stages. In contrast, redness tended to decrease over time, particularly in the Injection treatment by Day 21. Overall, Injection showed greater variation in color changes, while Control and Soaking remained relatively moderate. ^a–c^ Different letters show significantly different between ageing days (p<0.05). ^x–z^ Different letters show significantly different between treatments (p<0.05).

**Figure 2:**
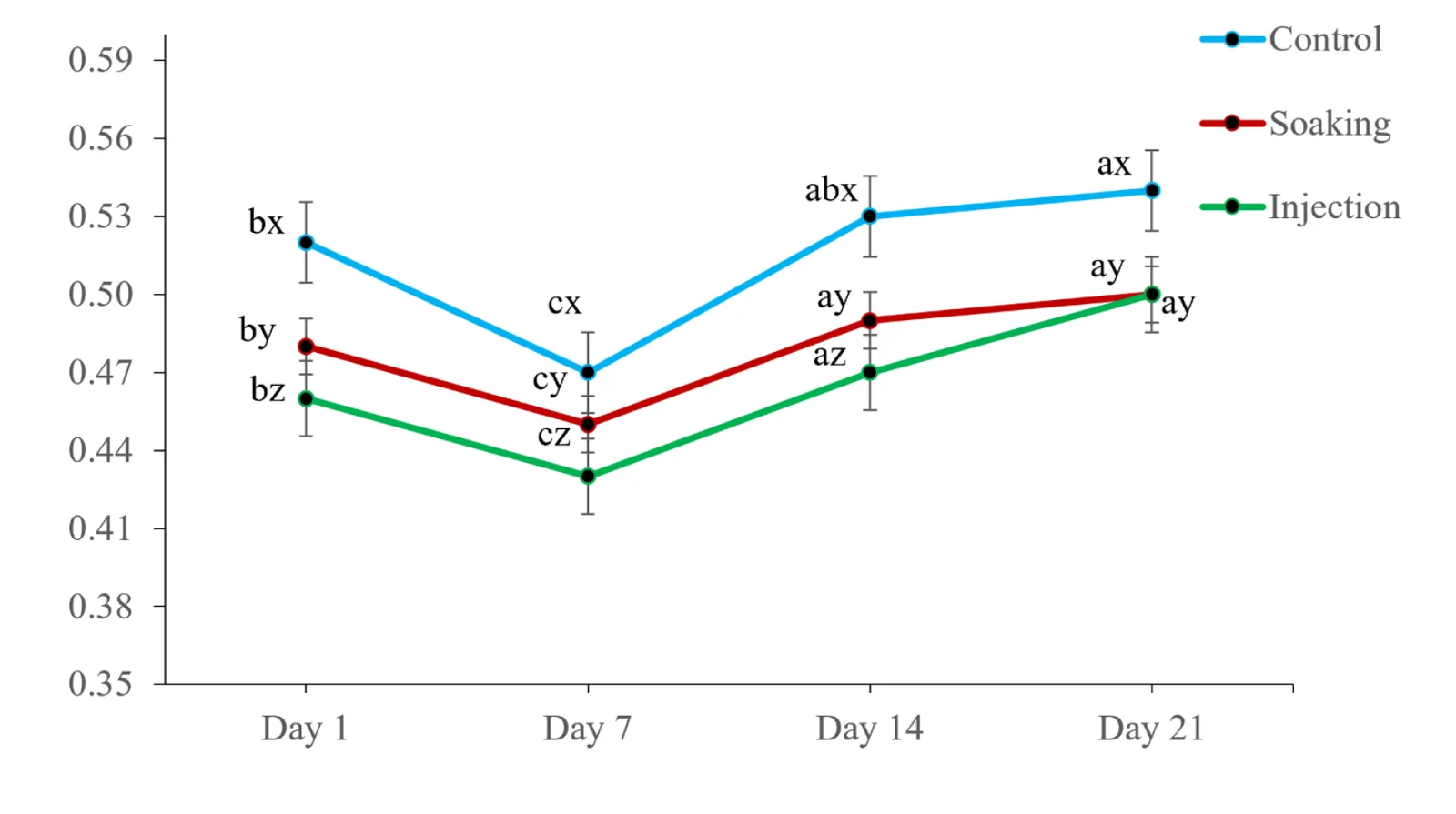
Effect of wet ageing with different marinating methods on lipid oxidation of goat meat during 21 d. Changes in measured values over time (Days 1, 7, 14, and 21) for Control, Soaking, and Injection treatments. Values decreased from Day 1 to Day 7 in all groups, followed by a gradual increase through Day 21. The Control group consistently showed the highest values, while Injection remained the lowest; Soaking was intermediate. ^a–c^ Means with an ageing day with different letters are significantly different (p<0.05). ^x–z^ Means with a treatment with different letters are significantly different (p<0.05).

**Figure 3:**
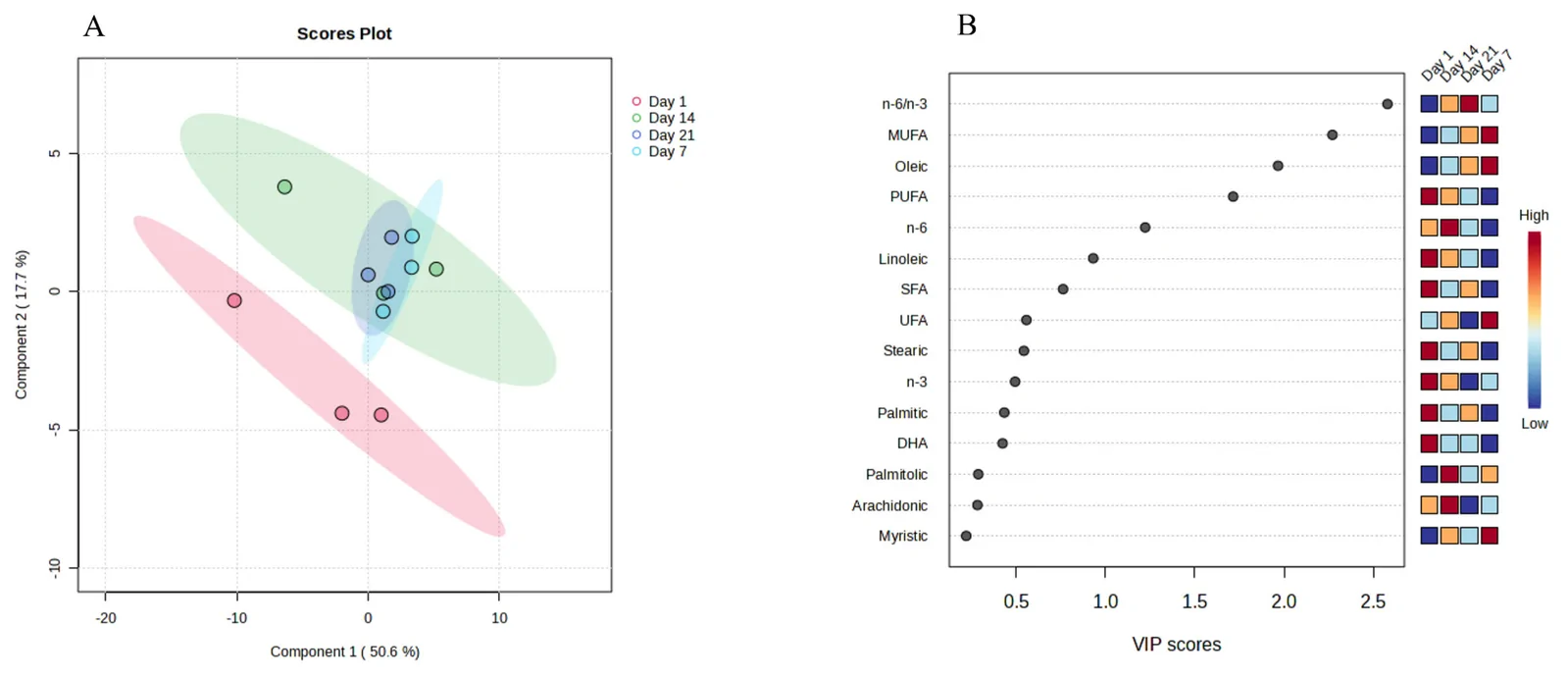
Result of (A) partial least square discriminant analysis (PLS-DA) and (B) variable important projection (VIP) score of changing fatty acids during ageing days. Multivariate analysis of sample distribution and variable importance across ageing periods (Days 1, 7, 14, and 21). The score plot (A) shows clear separation of samples by ageing time, with clustering indicating distinct characteristics at each stage. The VIP score plot (B) identifies the most influential variables contributing to this separation, with higher scores indicating greater importance. Color gradients represent relative variable contributions (low to high). MUFA, monounsaturated fatty acid; PUFA, polyunsaturated fatty acid; SFA, saturated fatty acid; UFA, unsaturated fatty acid; DHA, docosahexaenoic acid.

**Table 1:** Effect of wet ageing with different marinating methods on goat meat quality characteristics during 21 d

Items	Treatments	Ageing period (d)				SEM1)

		1	7	14	21	
Moisture (%)	Control	72.33^b^^z^	71.27^b^^z^	72.20^b^^y^	75.50^a^^x^	0.65
	Soaking	73.78^y^	73.50^y^	73.50^x^	74.00^y^	0.16
	Injection	74.89^a^^x^	72.50^c^^x^	74.00^b^^x^	73.67^b^^z^	0.22
	SEM2)	0.23	0.30	0.21	0.69	
Cooking loss (%)	Control	24.65^a^^y^	21.86^b^^y^	23.94^a^^y^	22.48^b^^y^	0.39
	Soaking	26.55^b^^x^	22.08^c^^x^	28.33^ab^^x^	28.60^a^^x^	0.57
	Injection	23.51^c^^z^	21.19^d^^y^	27.40^b^^x^	28.82^a^^x^	0.17
	SEM2)	0.32	0.46	0.56	0.22	
pH	Control	5.54^a^^x^	5.39^b^^x^	5.27^d^^y^	5.32^c^^x^	0.01
	Soaking	5.51^a^^y^	5.37^b^^y^	5.30^c^^x^	5.25^d^^y^	0.004
	Injection	5.55^a^^x^	5.34^b^^z^	5.18^c^^z^	4.96^d^^z^	0.01
	SEM2)	0.01	0.01	0.01	0.01	
Water holding capacity (%)	Control	91.51^c^^x^	96.16^a^^x^	94.64^b^^x^	96.38^a^^x^	0.11
	Soaking	89.57^c^^z^	93.90^a^^y^	94.12^a^^x^	90.74^b^^y^	0.23
	Injection	87.47^c^^y^	90.14^b^^z^	90.90^a^^y^	87.49^c^^z^	0.16
	SEM2)	0.08	0.21	0.20	0.18	
Total plate count (Log CFU/g)	Control	3.13^b^^x^	3.14^b^^x^	3.51^a^^x^	3.01^b^^x^	0.03
	Soaking	2.75^b^^z^	2.11^c^^y^	3.37^a^^y^	2.79^b^^y^	0.03
	Injection	2.96^a^^y^	2.36^b^^y^	2.93^a^^z^	2.58^b^^z^	0.03
	SEM2)	0.01	0.04	0.02	0.05	
Coliform count (Log CFU/g)	Control	2.83^b^^x^	2.32^d^^x^	3.31^a^^x^	2.58^c^^x^	0.05
	Soaking	2.15^c^^y^	1.84^d^^y^	3.26^a^^y^	2.30^b^^y^	0.05
	Injection	2.81^a^^x^	1.89^c^^y^	2.85^a^^z^	2.11^b^^z^	0.04
	SEM2)	0.02	0.06	0.02	0.06	

1) SEM: Standard error of the ageing period means.

2) SEM: Standard error of the treatment means.

a–d Means with a row with different letters are significantly different (p<0.05).

x–z Means with a column with different letters are significantly different (p<0.05).

**Table 2:** Effect of wet ageing with different marinating methods on texture profile analysis (TPA) and myofibrillar fragmentation index (MFI) of goat meat during 21 d

Items	Treatments	Ageing period (d)				SEM1)

		1	7	14	21	
TPA						
Hardness (kgf)	Control	4.77^a^^x^	3.37^b^^x^	3.20^b^^x^	3.06^b^^x^	0.16
	Soaking	3.77^a^^y^	2.54^b^^y^	2.49^b^^y^	2.07^c^^y^	0.09
	Injection	3.46^a^^y^	1.35^b^^z^	1.40^b^^z^	1.04^b^^z^	0.12
	SEM2)	0.18	0.10	0.12	0.04	
Chewiness (kgf)	Control	0.68^a^^z^	0.44^b^^z^	0.36^b^	0.34^b^^x^	0.24
	Soaking	0.63^a^^z^	0.41^b^^y^	0.37^b^	0.29^c^^y^	0.15
	Injection	0.75^a^^y^	0.41^b^^y^	0.33^b^^c^	0.23^c^^z^	0.12
	SEM2)	0.09	0.18	0.24	0.16	
Cohesiveness	Control	0.24^b^^y^	0.30^a^	0.29^a^	0.32^a^	0.05
	Soaking	0.30^x^	0.34	0.34	0.33	0.05
	Injection	0.32^x^	0.34	0.33	0.35	0.03
	SEM2)	0.02	0.03	0.10	0.07	
Springiness	Control	0.55	0.6	0.57	0.58	0.05
	Soaking	0.61^b^	0.85^a^	0.72^b^	0.61^b^	0.04
	Injection	0.57	0.81	0.64	0.61	0.06
	SEM2)	0.03	0.07	0.04	0.05	
Gumminess (kgf)	Control	0.58^a^^y^	0.45^b^^y^	0.43^b^	0.32^c^	0.04
	Soaking	0.47^a^^z^	0.36^b^^z^	0.39^b^	0.27^c^	0.02
	Injection	0.62^a^^y^	0.35^b^^z^	0.37^b^	0.25^c^	0.01
	SEM2)	0.13	0.12	0.17	0.14	
MFI						
MFI (%)	Control	111.93^b^^y^	112.87^b^^y^	121.55^a^^z^	122.23^a^^y^	3.22
	Soaking	133.47^b^^x^	136.53^ab^^x^	138.45^ab^^y^	143.75^a^^x^	2.14
	Injection	124.53^c^^x^	136.93^b^^x^	144.04^a^^x^	144.90^a^^x^	0.62
	SEM2)	3.52	1.01	1.77	1.99	

Springiness was expressed as a ratio calculated from the distance recovered after the first compression cycle.

1) SEM: Standard error of the ageing period means.

2) SEM: Standard error of the treatment means.

a–c Means with a row with different letters are significantly different (p<0.05).

x–z Means with a column with different letters are significantly different (p<0.05).

**Table 3:** Change in the fatty acid composition in wet ageing goat meat with different marinating methods during 21 d

Items (%)	Treatments	Ageing period (d)				SEM1)

		1	7	14	21	
Capric (10:0)	Control	0.08	0.07	0.07^z^	0.07^z^	0.003
	Soaking	0.09	0.08	0.08^z^	0.10^y^	0.004
	Injection	0.09^b^	0.08^b^	0.11^a^^y^	0.09^b^^y^	0.007
	SEM2)	0.005	0.005	0.012	0.010	
Lauric (12:0)	Control	0.14^c^^z^	0.17^b^^y^	0.23^a^^y^	0.16^b^^y^	0.019
	Soaking	0.18^a^^y^	0.06^c^^z^	0.07^c^^z^	0.14^b^^z^	0.029
	Injection	0.13^b^^z^	0.26^a^^x^	0.28^a^^x^	0.13^b^^z^	0.039
	SEM2)	0.053	0.048	0.065	0.047	
Myristic (14:0)	Control	2.24^c^	3.00^a^^y^	2.94^a^^y^	2.70^b^^x^	0.173
	Soaking	2.22^a^	1.52^b^^z^	1.56^b^^z^	2.18^a^^y^	0.191
	Injection	2.23^b^	3.18^a^^y^	2.72^b^^y^	2.06^c^^z^	0.253
	SEM2)	0.005	0.526	0.428	0.197	
Palmitic (16:0)	Control	22.79	23.66^y^	23.92^y^	22.45	0.348
	Soaking	22.47^a^	18.22^b^^z^	18.40^b^^z^	22.97^a^	1.276
	Injection	23.08	24.04^y^	23.65^y^	22.38	0.361
	SEM2)	0.177	1.881	1.797	0.186	
Palmitoleic (16:1)	Control	1.67^b^^z^	2.68^a^	2.77^a^	2.57^a^^y^	0.253
	Soaking	2.51^a^^y^	2.55^a^	2.70^a^	1.88^a^^z^	0.182
	Injection	1.98^b^^z^	2.72^a^	2.49^a^	1.74^b^^z^	0.227
	SEM2)	0.245	0.051	0.085	0.256	
Stearic (18:0)	Control	11.98^a^^y^	9.27^b^^z^	9.75^b^^z^	9.79^b^^z^	0.607
	Soaking	9.69^c^^z^	10.93^b^^y^	11.04^a^^y^	11.41^a^^y^	0.373
	Injection	11.63^a^^y^	10.10^b^^y^	9.89^c^^z^	11.67^a^^y^	0.479
	SEM2)	0.713	0.482	0.410	0.589	
Oleic (18:1)	Control	45.72^b^^y^	47.08^a^^y^	45.74^b^^y^	45.96^b^	0.325
	Soaking	39.90^c^^z^	47.34^a^^y^	48.58^a^^x^	46.37^b^	1.937
	Injection	47.32^a^^x^	45.67^b^^z^	40.20^c^^z^	45.44^b^	1.545
	SEM2)	2.258	0.520	2.465	0.269	
Linoleic (18:2)	Control	6.63^a^^z^	5.95^b^^z^	6.07^b^^z^	6.77^a^^z^	0.204
	Soaking	10.24^a^^y^	7.33^b^^y^	6.51^c^^z^	6.49^c^^z^	0.887
	Injection	5.53^c^^z^	5.63^c^^z^	9.13^a^^y^	7.23^b^^y^	0.844
	SEM2)	1.422	0.521	0.957	0.217	
Linolenic (18:3)	Control	0.36^c^	0.40^bc^^z^	0.47^a^^y^	0.42^b^^y^	0.023
	Soaking	0.32^b^	0.51^a^^y^	0.53^a^^x^	0.30^b^^z^	0.060
	Injection	0.36^b^	0.47^a^^y^	0.32^bc^^z^	0.27^c^^z^	0.041
	SEM2)	0.012	0.031	0.061	0.044	
Eicosadienoic (20:2)	Control	0.07	0.06	0.05	0.06	0.004
	Soaking	0.08	0.06	0.06	0.07	0.004
	Injection	0.06	0.05	0.07	0.07	0.005
	SEM2)	0.006	0.004	0.004	0.002	
Eicosatrienoic (20:3)	Control	0.25^z^	0.23^z^	0.24^z^	0.27	0.008
	Soaking	0.40^a^^y^	0.28^b^^y^	0.24^b^^z^	0.25^b^	0.036
	Injection	0.20^b^^z^	0.23^b^^z^	0.34^a^^y^	0.29^a^	0.031
	SEM2)	0.058	0.016	0.034	0.011	
Arachidonic (20:4)	Control	2.97^b^^z^	3.03^b^^z^	3.11^b^^z^	3.81^a^^y^	0.196
	Soaking	5.68^a^^y^	4.86^a^^y^	4.14^b^^y^	2.85^c^^z^	0.600
	Injection	2.63^c^^z^	2.91^c^^z^	4.84^a^^y^	3.33^b^^y^	0.493
	SEM2)	0.967	0.632	0.503	0.278	
EPA (20:5)	Control	0.29^a^^z^	0.15^b^^z^	0.20^b^^z^	0.17^b^^z^	0.031
	Soaking	0.47^a^^y^	0.18^b^^y^	0.14^b^^z^	0.27^b^^y^	0.074
	Injection	0.20^c^^z^	0.18^c^^y^	0.42^a^^y^	0.34^b^^x^	0.056
	SEM2)	0.079	0.009	0.084	0.048	
DHA (22:6)	Control	0.57^a^^y^	0.06^b^	0.05^b^	0.07^b^	0.127
	Soaking	0.92^a^^x^	0.07^b^	0.06^b^	0.06^b^	0.214
	Injection	0.45^a^^z^	0.04^b^	0.09^b^	0.07^b^	0.095
	SEM2)	0.141	0.007	0.013	0.003	
Nervonic (24:1)	Control	0.28^b^^z^	0.31^b^^z^	0.27^b^^z^	0.40^a^^y^	0.029
	Soaking	0.46^a^^y^	0.57^a^^y^	0.50^a^^x^	0.29^b^^z^	0.060
	Injection	0.30^b^^z^	0.27^b^^z^	0.38^a^^y^	0.31^b^^z^	0.025
	SEM2)	0.058	0.093	0.066	0.035	
SFA	Control	37.22^a^^y^	36.17^b^^y^	36.90^b^^y^	35.17^c^	0.455
	Soaking	34.66^z^^a^	30.82^z^^b^	31.16^b^^z^	36.80^a^	1.438
	Injection	37.15^a^^y^	37.65^a^^y^	36.64^b^^y^	36.33^b^	0.290
	SEM2)	0.845	2.076	1.874	0.486	
UFA	Control	58.81^b^^z^	59.97^ab^^z^	58.96^b^^z^	60.51^a^	0.407
	Soaking	60.97^b^^x^	63.76^a^^y^	63.47^a^^y^	58.83^c^	1.159
	Injection	59.03^a^^y^	58.17^b^^z^	58.27^b^^z^	59.08^a^	0.242
	SEM2)	0.688	1.649	1.631	0.524	
MUFA	Control	47.67^c^^y^	50.08^a^^y^	48.78^b^^y^	48.93^b^	0.492
	Soaking	42.87^c^^z^	50.47^b^^y^	51.79^a^^x^	48.54^b^	1.965
	Injection	49.60^a^^x^	48.66^a^^z^	43.07^c^^z^	47.49^b^	1.445
	SEM2)	2.003	0.549	2.560	0.431	
PUFA	Control	11.14^a^^y^	9.89^b^^z^	10.18^b^^z^	11.58^a^	0.397
	Soaking	18.10^a^^x^	13.29^b^^y^	11.68^b^^z^	10.29^b^	1.702
	Injection	9.43^c^^z^	9.51^c^^z^	15.20^a^^y^	11.59^b^	1.353
	SEM2)	2.655	1.205	1.491	0.434	

1) SEM: Standard error of the ageing period means.

2) SEM: Standard error of the treatment means.

a–c Means with a row with different letters are significantly different (p<0.05).

x–z Means with a column with different letters are significantly different (p<0.05).

EPA, eicosapentaenoic acid; DHA, docosahexaenoic acid; SFA, saturated fatty acid; UFA, unsaturated fatty acid; MUFA, monounsaturated fatty acid; PUFA, polyunsaturated fatty acid.

**Table 4:** Effect of wet ageing with different marination methods on nutritional quality indices of goat meat during 21 d

Items (%)	Treatments	Ageing days				SEM1)

		1	7	14	21	
PUFA/SFA	Control	0.30^z^	0.27^z^	0.28^z^	0.33	0.013
	Soaking	0.52^a^^y^	0.43^b^^y^	0.37^b^^y^	0.28^c^	0.016
	Injection	0.25^c^^z^	0.25^c^^z^	0.41^a^^y^	0.32^b^	0.021
	SEM2)	0.041	0.036	0.016	0.013	
n-6/n-3	Control	6.62^c^^z^	10.57^b^^y^	9.69^b^^z^	11.43^a^	1.049
	Soaking	7.60^b^^y^	11.79^a^^x^	11.11^a^^y^	10.69^a^	0.927
	Injection	6.79^c^^z^	9.33^bc^^z^	12.02^a^^x^	11.07^b^	1.148
	SEM2)	0.304	0.714	0.679	0.216	
n-6	Control	9.68^b^^z^	9.04^b^^z^	9.23^b^^z^	10.65^a^^y^	0.360
	Soaking	16.00^a^^y^	12.25^b^^y^	10.71^c^^z^	9.41^c^^z^	1.426
	Injection	8.22^c^^z^	8.59^c^^z^	14.04^a^^y^	10.63^b^^y^	1.332
	SEM2)	2.390	1.156	1.423	0.412	
n-3	Control	1.46^a^^z^	0.85^b^^z^	0.95^b^^z^	0.93^b^	0.139
	Soaking	2.10^a^^y^	1.04^b^^y^	0.96^b^^z^	0.88^b^	0.288
	Injection	1.21^z^	0.92^z^	1.17^y^	0.96	0.074
	SEM2)	0.266	0.054	0.070	0.024	
AI	Control	0.54^b^	0.60^a^^y^	0.61^a^^y^	0.55^b^	0.017
	Soaking	0.52^a^	0.38^b^^z^	0.39^b^^z^	0.54^a^	0.042
	Injection	0.54^b^	0.64^a^^y^	0.60^a^^y^	0.52^b^	0.026
	SEM2)	0.009	0.079	0.071	0.009	
TI	Control	1.11^y^	1.12^y^	1.15^y^	1.07	0.016
	Soaking	0.96^b^^z^	0.89^b^^z^	0.91^b^^z^	1.15^a^	0.061
	Injection	1.13^y^	1.1^y^	1.13^y^	1.13	0.015
	SEM2)	0.055	0.090	0.077	0.025	
HH	Control	2.26	2.13^z^	2.07^z^	2.27	0.050
	Soaking	2.33^b^	3.06^a^^y^	3.00^a^^y^	2.24^b^	0.216
	Injection	2.23	2.02^z^	2.09^z^	2.32	0.068
	SEM2)	0.029	0.329	0.309	0.023	

1) SEM: Standard error of the ageing period means.

2) SEM: Standard error of the treatment means.

a–c Means with a row with different letters are significantly different (p<0.05).

x–z Means with a column with different letters are significantly different (p<0.05).

PUFA, polyunsaturated fatty acid; SFA, saturated fatty acid; AI, atherogenicity index; TI, thrombogenicity index; HH, hypocholesterolemic/hypercholesterolemic.

## Data Availability

Upon reasonable request, the datasets of this study can be available from the corresponding author.
